# Type 2 diabetes, hepatic decompensation, and hepatocellular carcinoma in patients with non-alcoholic fatty liver disease: an individual participant-level data meta-analysis

**DOI:** 10.1016/S2468-1253(23)00157-7

**Published:** 2023-07-04

**Authors:** Daniel Q Huang, Nabil Noureddin, Veeral Ajmera, Maral Amangurbanova, Ricki Bettencourt, Emily Truong, Tolga Gidener, Harris Siddiqi, Abdul M Majzoub, Tarek Nayfeh, Nobuharu Tamaki, Namiki Izumi, Masato Yoneda, Atsushi Nakajima, Ramazan Idilman, Mesut Gumussoy, Digdem Kuru Oz, Ayse Erden, Alina M Allen, Mazen Noureddin, Rohit Loomba

**Affiliations:** NAFLD Research Center, Division of Gastroenterology and Hepatology, Department of Medicine, University of California San Diego, La Jolla, CA, USA; Department of Medicine, Yong Loo Lin School of Medicine, National University of Singapore, Singapore; Division of Gastroenterology and Hepatology, Department of Medicine, National University Health System, Singapore; NAFLD Research Center, Division of Gastroenterology and Hepatology, Department of Medicine, University of California San Diego, La Jolla, CA, USA; NAFLD Research Center, Division of Gastroenterology and Hepatology, Department of Medicine, University of California San Diego, La Jolla, CA, USA; NAFLD Research Center, Division of Gastroenterology and Hepatology, Department of Medicine, University of California San Diego, La Jolla, CA, USA; NAFLD Research Center, Division of Gastroenterology and Hepatology, Department of Medicine, University of California San Diego, La Jolla, CA, USA; Department of Medicine, Cedars-Sinai Medical Center, Los Angeles, CA, USA; Division of Gastroenterology and Hepatolog, Mayo Clinic, Rochester, MN, USA; NAFLD Research Center, Division of Gastroenterology and Hepatology, Department of Medicine, University of California San Diego, La Jolla, CA, USA; Evidence-Based Practice Center, Mayo Clinic, Rochester, MN, USA; Division of Gastroenterology and Hepatology, Department of Medicine, University of Missouri, Columbia, MO, USA; Evidence-Based Practice Center, Mayo Clinic, Rochester, MN, USA; NAFLD Research Center, Division of Gastroenterology and Hepatology, Department of Medicine, University of California San Diego, La Jolla, CA, USA; Department of Gastroenterology and Hepatology, Musashino Red Cross Hospital, Tokyo, Japan; Department of Gastroenterology and Hepatology, Musashino Red Cross Hospital, Tokyo, Japan; Department of Gastroenterology and Hepatology, Yokohama City University, Yokohama, Japan; Department of Gastroenterology and Hepatology, Yokohama City University, Yokohama, Japan; Department of Gastroenterology, School of Medicine, Ankara University, Ankara, Turkey; Department of Gastroenterology, School of Medicine, Ankara University, Ankara, Turkey; Department of Radiology, School of Medicine, Ankara University, Ankara, Turkey; Department of Radiology, School of Medicine, Ankara University, Ankara, Turkey; Division of Gastroenterology and Hepatology, Mayo Clinic, Rochester, MN, USA; Houston Methodist Transplant Center, Houston, TX, USA; Houston Liver Institute, Houston, TX, USA; NAFLD Research Center, Division of Gastroenterology and Hepatology, Department of Medicine, University of California San Diego, La Jolla, CA, USA

## Abstract

**Background:**

Data are scarce regarding the development of hepatic decompensation in patients with non-alcoholic fatty liver disease (NAFLD) with and without type 2 diabetes. We aimed to assess the risk of hepatic decompensation in people with NAFLD with and without type 2 diabetes.

**Methods:**

We did a meta-analysis of individual participant-level data from six cohorts in the USA, Japan, and Turkey. Included participants had magnetic resonance elastography between Feb 27, 2007, and June 4, 2021. Eligible studies included those with liver fibrosis characterisation by magnetic resonance elastography, longitudinal assessment for hepatic decompensation and death, and included adult patients (aged ≥18 years) with NAFLD, for whom data were available regarding the presence of type 2 diabetes at baseline. The primary outcome was hepatic decompensation, defined as ascites, hepatic encephalopathy, or variceal bleeding. The secondary outcome was the development of hepatocellular carcinoma. We used competing risk regression using the Fine and Gray subdistribution hazard ratio (sHR) to compare the likelihood of hepatic decompensation in participants with and without type 2 diabetes. Death without hepatic decompensation was a competing event.

**Findings:**

Data for 2016 participants (736 with type 2 diabetes; 1280 without type 2 diabetes) from six cohorts were included in this analysis. 1074 (53%) of 2016 participants were female with a mean age of 57·8 years (SD 14·2) years and BMI of 31·3 kg/m^2^ (SD 7·4). Among 1737 participants (602 with type 2 diabetes and 1135 without type 2 diabetes) with available longitudinal data, 105 participants developed hepatic decompensation over a median follow-up time of 2·8 years (IQR 1·4–5·5). Participants with type 2 diabetes had a significantly higher risk of hepatic decompensation at 1 year (3·37% [95% CI 2·10–5·11] *vs* 1·07% [0·57–1·86]), 3 years (7·49% [5·36–10·08] *vs* 2·92% [1·92–4·25]), and 5 years (13·85% [10·43–17·75] *vs* 3·95% [2·67–5·60]) than participants without type 2 diabetes (p<0·0001). After adjustment for multiple confounders (age, BMI, and race), type 2 diabetes (sHR 2·15 [95% CI 1·39–3·34]; p=0·0006) and glycated haemoglobin (1·31 [95% CI 1·10–1·55]; p=0·0019) were independent predictors of hepatic decompensation. The association between type 2 diabetes and hepatic decompensation remained consistent after adjustment for baseline liver stiffness determined by magnetic resonance elastography. Over a median follow-up of 2·9 years (IQR 1·4–5·7), 22 of 1802 participants analysed (18 of 639 with type 2 diabetes and four of 1163 without type 2 diabetes) developed incident hepatocellular carcinoma. The risk of incident hepatocellular carcinoma was higher in those with type 2 diabetes at 1 year (1·34% [95% CI 0·64–2·54] *vs* 0·09% [0·01–0·50], 3 years (2·44% [1·36–4·05] *vs* 0·21% [0·04–0·73]), and 5 years (3·68% [2·18–5·77] *vs* 0·44% [0·11–1·33]) than in those without type 2 diabetes (p<0·0001). Type 2 diabetes was an independent predictor of hepatocellular carcinoma development (sHR 5·34 [1·67–17·09]; p=0·0048).

**Interpretation:**

Among people with NAFLD, the presence of type 2 diabetes is associated with a significantly higher risk of hepatic decompensation and hepatocellular carcinoma.

**Funding:**

National Institute of Diabetes and Digestive and Kidney Diseases.

## Introduction

Non-alcoholic fatty liver disease (NAFLD) affects a third of the global adult population and is a major cause of liver-related morbidity and mortality.^1-6^ NAFLD includes non-alcoholic fatty liver and non-alcoholic steatohepatitis (NASH), the inflammatory form of NAFLD that can progress to fibrosis and hepatocellular carcinoma.^7-14^ Nearly 10% of the global population has type 2 diabetes, more than a third of individuals with type 2 diabetes have NASH, and around one in six individuals with type 2 diabetes have advanced fibrosis.^15^-^17^

Previous studies have shown that type 2 diabetes is associated with hepatic decompensation among people with cirrhosis, hepatitis C virus, and heavy alcohol consumption.^8-25^ However, the risk of hepatic decompensation (development of ascites, hepatic encephalopathy, or variceal bleeding) among individuals with NAFLD with and without type 2 diabetes has not been systematically assessed. Therefore, we aimed to examine the association between type 2 diabetes and liver-related outcomes through an individual participant data meta-analysis, accounting for competing risks.

## Methods

### Search strategy and selection criteria

We previously published an individual participant data meta-analysis^26^ to examine the association between liver stiffness on magnetic resonance elastography and liver-related outcomes. This individual participant-level data meta-analysis is an extension of the previous study and focuses on the association between type 2 diabetes and liver-related outcomes. A medical librarian conducted a systematic literature search of several databases from inception to April 24, 2023. The databases included MEDLINE on Ovid, Evidence-Based Medicine Reviews, the Cochrane Central Register of Controlled Trials, Cochrane Database of Systematic Reviews, Scopus, Web of Science, and Embase (details of the search strategies are provided in the appendix pp 2-3).^26^ Additionally, experts in the field were consulted to identify additional published and unpublished primary studies. Two investigators (AMM and TN) independently reviewed the titles and abstracts of all citations identified by the search, and full-text manuscripts for potentially relevant articles were revised. Disagreements were resolved by consensus and a third reviewer (DQH) if needed.

Studies were included if they met the following inclusion criteria: (1) characterisation of liver stiffness by magnetic resonance elastography, (2) longitudinal assessment for hepatic decompensation, defined as any of the following: ascites, hepatic encephalopathy, or variceal bleeding, and death, (3) adult patients (aged ≥18 years) with NAFLD, and (4) availability of data for the presence of type 2 diabetes at baseline. NAFLD was defined as hepatic steatosis on imaging or historical liver biopsy without clinically significant alcohol consumption, secondary causes of hepatic steatosis, and other chronic underlying liver disease including viral hepatitis.

Six investigators were approached to obtain individual participant-level data. All six investigators responded and provided these data.

This individual participant data meta-analysis was conducted according to PRISMA-individual participant data guidelines. The study was approved by the institutional review board at each participating site, and analysis used de-identified data.

### Data analysis

The following data from each included study were requested, extracted, and systematically checked. No major issues with data quality were identified when checking individual participant data. Demographic data were retrieved from consecutive participants, including age at the time of magnetic resonance elastography, sex, race or ethnicity, and BMI. Data for metabolic comorbidities were recorded, including hypertension, hyperlipidaemia, and type 2 diabetes. Results from the following biochemical tests were requested: albumin, glycated haemoglobin (HbA_1c_), alanine aminotransferase, aspartate aminotransferase, total bilirubin, alkaline phosphatase, fasting lipid panel, platelet count, international normalised ratio, sodium, and creatinine. The presence of type 2 diabetes at baseline was based on the clinical practice recommendations from the American Diabetes Association and included any of the following criteria: HbA_1c_ greater than or equal to 6·5%; fasting plasma glucose greater than or equal to 126 mg/dL (7·0 mmol/L); plasma glucose greater than or equal to 200 mg/dL (11·1 mmol/L); or medical diagnosis of type 2 diabetes or use of medications to treat type 2 diabetes.^27^

NAFLD was diagnosed on imaging and clinical criteria consistent with the American Association for the Study of Liver Diseases (AASLD) NAFLD Practice Guidance.^6^ Liver stiffness data using two dimensional magnetic resonance elastography were obtained.

The risk of bias was assessed by two independent investigators using the QUADAS-2 tool, which consists of four key domains covering patient selection, index test, reference standard, and flow of patients through the study and timing of the index tests and reference standard.^28^

### Outcomes

The primary outcome was hepatic decompensation, defined as any of the following: ascites, hepatic encephalopathy, or variceal bleeding, assessed by the local site investigator. Ascites was defined per AASLD guidance by imaging or physical exam.^29^ Hepatic encephalopathy was defined as brain dysfunction caused by liver dysfunction or portosystemic shunting, as per practice guidelines.^30^

The secondary outcome was the development of hepatocellular carcinoma, diagnosed by the Liver Imaging Reporting and Data System for definite hepatocellular carcinoma, Liver Reporting and Data Systems category 5, or histology.

The index date was defined as the date of the baseline magnetic resonance elastography. All participants were followed until death or the last clinical encounter and assessed by chart review. Participants with hepatic decompensation or hepatocellular carcinoma diagnosed within 1 month of the baseline magnetic resonance elastography were excluded from the analyses for incident outcomes.

### Statistical analysis

Demographic, laboratory, imaging, and outcome data were presented as mean (SD) or median (IQR) for continuous variables, and as numbers and percentages for categorical variables. Baseline categorical variables were compared using the χ^2^ test, and continuous data were compared using the Student’s *t*-test for parametric data and the Wilcoxon rank-sum tests for non-parametric data. Cumulative incidence curves were generated to evaluate the risks of hepatic decompensation and hepatocellular carcinoma, after accounting for competing risks of death without hepatic decompensation, and death without hepatocellular carcinoma, respectively. Participants were censored at the time of death. We did univariable and multivariable logistic regression to determine the odds ratios (ORs) for the presence of hepatic decompensation at baseline. We did competing risk regression using the Fine and Gray subdistribution hazard ratio (sHR)^31^ with multivariable adjustment to estimate the likelihood of hepatic decompensation after accounting for the competing risk of death without hepatic decompensation, because of the elevated risk of non-liver related mortality in people with NAFLD.^32-36^ Similarly, the Fine and Gray sHR with multivariable adjustment was used to determine the likelihood of hepatocellular carcinoma after accounting for the competing risk of death without hepatocellular carcinoma. We identified potential confounders based on previous reports in the literature^36-40^ and selected a minimally sufficient set of confounders using a causal-directed acyclic graph (appendix p 20). The final minimally sufficient set of confounders comprised age, race or ethnicity, and BMI. The proportional hazards assumption was assessed for categorical covariates using graphs of the Kaplan-Meier estimates of the survival function and all seemed to adhere to the proportional hazards assumption. Continuous covariates were assessed by plotting scaled Schoenfeld residuals versus functions of time. All of the smoothed LOESS plots were mostly flat at 0 suggesting that the coefficients did not change over time and that the proportional hazards assumptions held. We explored the functional form of the covariates creating the smooth of a scatter plot of the Martingale residuals from a null model versus each covariate individually. The plots at the higher smoothing parameter values had similar shapes, which seemed to indicate a linear effect. Prespecified subgroup analyses were done for liver stiffness measurements on magnetic resonance elastography of 5 kPa or more and less than 5 kPa, corresponding to the thresholds recommended by the AASLD guidance to identify cirrhosis.^6^ All statistical analyses were performed using SAS (version 9.4), and p values of less than 0·05 were considered to indicate statistically significant differences.

### Role of the funding source

The funder of the study had no role in study design, data collection, data analysis, data interpretation, or writing of the report.

## Results

Six studies were included in this individual participant-level data meta-analysis, of which five^41-45^ have been published, and one is unpublished (appendix p 21). All included studies were retrospective and were done at tertiary centres, with patients recruited at clinics and through newspaper advertisements. Three studies were from the USA,^41,42,45^ two from Japan,^43,44^ and one from Turkey. The risk of bias assessment is shown in the appendix (p 4).

2016 participants (1074 [53·3%] female; 942 [46·7%] male) who underwent magnetic resonance elastography between Feb 27, 2007 and June 4, 2021, and had data indicating the presence or absence of type 2 diabetes were included in this study. The mean age of participants was 57·8 years (SD 14·2) and the mean BMI was 31·3 kg/m^2^ (SD 7·4; table 1). At baseline, 736 participants had type 2 diabetes and 1280 did not. Participants with type 2 diabetes at baseline were older and had higher BMI, HbA_1c_, aspartate aminotransferase, fibrosis-4 index (FIB-4), NAFLD fibrosis score, and liver stiffness on magnetic resonance elastography than participants without type 2 diabetes at baseline (table 1).

114 (5·7%) of 2016 participants (70 participants with type 2 diabetes; 44 participants without type 2 diabetes) had hepatic decompensation at baseline (or within 30 days of the index date); 159 events [ascites, n=91; hepatic encephalopathy, n=43; variceal haemorrhages, n=25). The presence of type 2 diabetes was associated with hepatic decompensation at baseline (OR 2·95 [95% CI 2·00–4·36]; p<0·0001). The association of type 2 diabetes with hepatic decompensation at baseline remained consistent on multivariable analysis (adjusted OR 3·08 [95% CI 1·98–4·78]; p<0·0001) even after adjustment for age, BMI, and race or ethnicity (appendix p 5).

**Figure 1:**
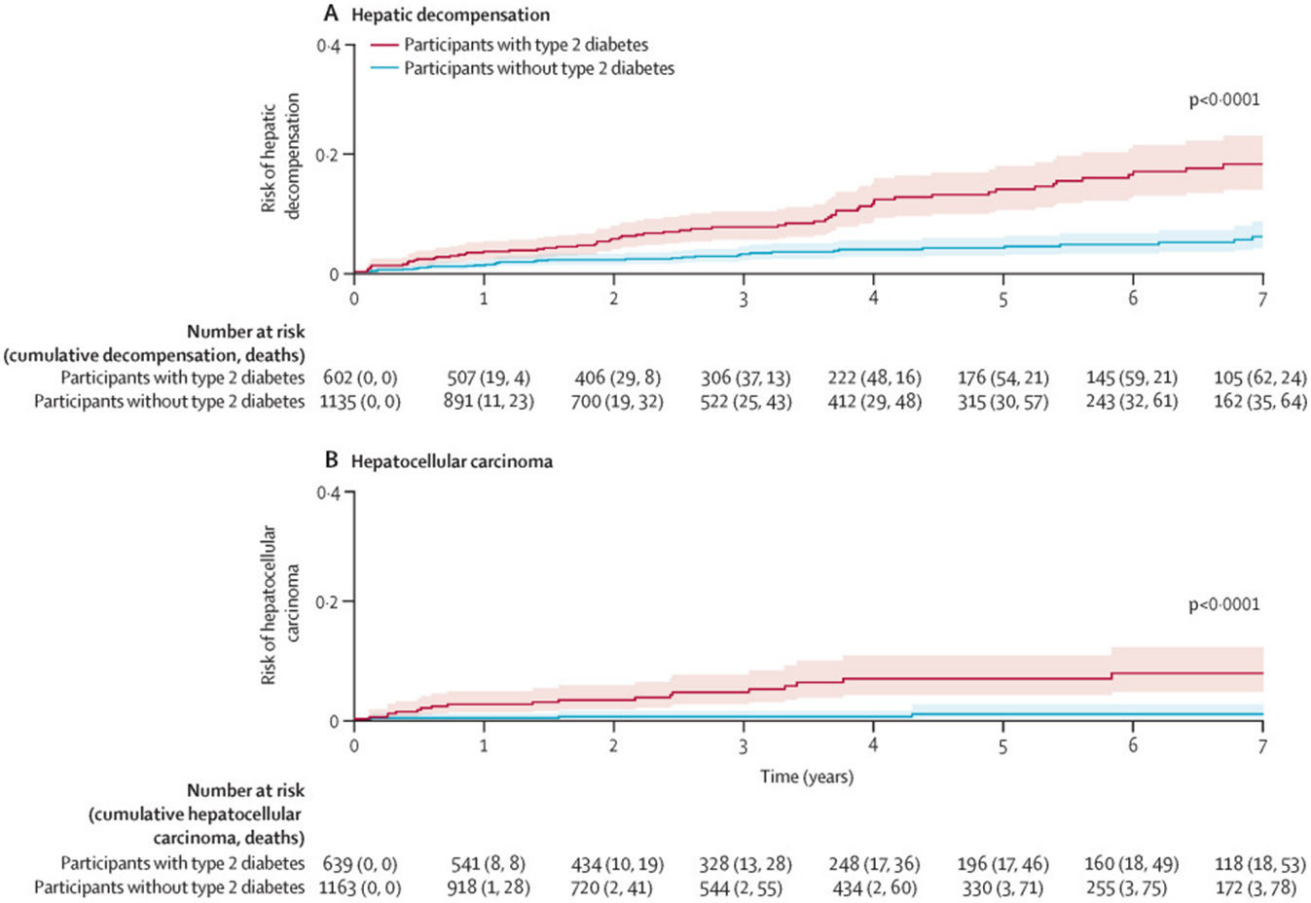
Risk of hepatic decompensation (A) and hepatocellular carcinoma (B) in participants with non-alcoholic fatty liver disease, with death as a competing risk, stratified by type 2 diabetes status Hepatic decompensation was defined as ascites, hepatic encephalopathy, or variceal bleeding. Cumulative cases of decompensation, deaths, and hepatocellular carcinoma are shown until year 7 of follow-up. Graphs are truncated as the number of participants at risk was low after 7 years.

After excluding participants with hepatic decompensation at baseline (n=114), and those without follow-up data (n=165; appendix p 6), 1737 participants (602 with type 2 diabetes and 1135 without type 2 diabetes) were included in the analysis for incident hepatic decompensation. Over a median follow-up of 2·8 years (IQR 1·4–5·5), 205 (11·8%) of 1737 participants developed hepatic decompensation or died without hepatic decompensation; 105 participants (68 with type 2 diabetes, 37 without type 2 diabetes) developed hepatic decompensation (155 events [ascites, n=87; hepatic encephalopathy, n=50; variceal bleeding, n=18), while 100 participants (31 with type 2 diabetes, 69 without type 2 diabetes) died without previous hepatic decompensation. The total number of person-years at risk in participants with type 2 diabetes were 2269 and 3984 among participants without type 2 diabetes. The 1-year, 3-year, and 5-year risks of incident hepatic decompensation were 3·37% (95% CI 2·10–5·11), 7·49% (5·36–10·08), and 13·85% (10·43–17·75) in participants with type 2 diabetes at baseline, and 1·07% (0·57–1·86), 2·92% (1·92–4·25), and 3·95% (2·67–5·60) in participants without type 2 diabetes at baseline (p<0·0001; figure 1A).

The presence of type 2 diabetes at baseline was associated with the development of incident hepatic decompensation (sHR 3·29 [95% CI 2·21–4·90]; p<0·0001). Type 2 diabetes remained an independent predictor of incident hepatic decompensation (2·15 [1·39–3·34]; p=0·0006) after adjustment for age, BMI, and race or ethnicity and accounting for competing risks (table 2). HbA_1c_ was an independent predictor for incident hepatic decompensation (1·31 [1·10–1·55]; p=0·0019), after adjustment for age, BMI, and race or ethnicity (appendix p 7).

After excluding participants with hepatocellular carcinoma at baseline (n=47), and those without follow-up data (n=167), 1802 participants (639 with type 2 diabetes and 1163 without type 2 diabetes) were included in the analysis for incident hepatocellular carcinoma. Over a median follow-up of 2·9 years (IQR 1·4–5·7), 22 participants (18 with type 2 diabetes; four without type 2 diabetes) developed incident hepatocellular carcinoma. The total number of person-years at risk for hepatocellular carcinoma was 2472 person-years in participants with type 2 diabetes and 4139 person-years in participants without type 2 diabetes. The 1-year, 3-year, and 5-year risks of incident hepatocellular carcinoma were 1·34% (95% CI 0·64–2·54), 2·44% (1·36–4·05), and 3·68% (2·18–5·77) in participants with type 2 diabetes at baseline and 0·09% (0·01–0·50), 0·21% (0·04–0·73), and 0·44% (0·11–1·33) in participants without type 2 diabetes at baseline (p<0·0001; figure 1B).

Type 2 diabetes at baseline was associated with the development of incident hepatocellular carcinoma (sHR 7·72 [95% CI 2·61–22·87]; p=0·0002). Type 2 diabetes remained an independent predictor of incident hepatocellular carcinoma (5·34 [1·67–17·09]; p=0·0048) after adjustment for age, BMI, and race or ethnicity and accounting for competing risks (table 3). HbA_1c_ was an independent predictor of incident hepatocellular carcinoma (1·32 [1·02–1·71]; p=0·034), after adjusting for confounders (appendix p 8).

**Figure 2:**
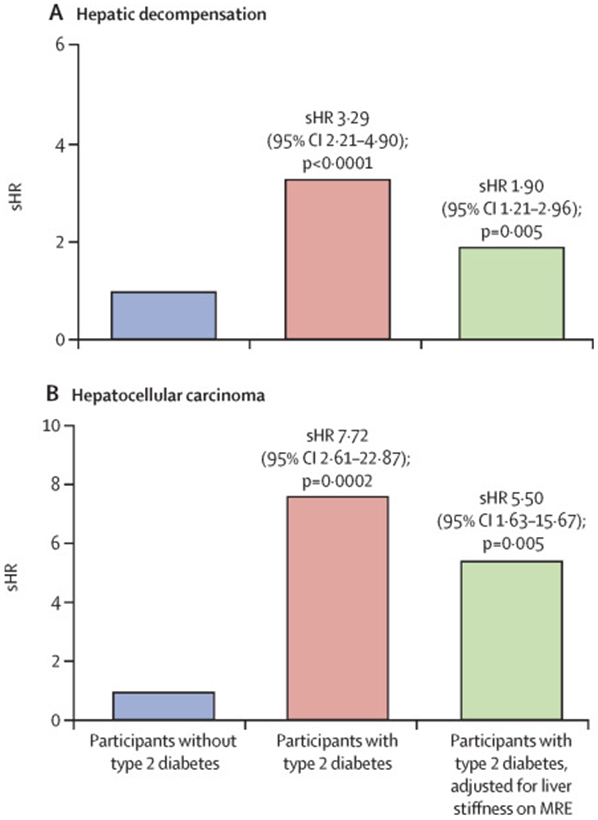
sHR for hepatic decompensation (A) and hepatocellular carcinoma (B) sHR=subdistribution=hazard ratio. MRE=magnetic resonance elastography.

Type 2 diabetes remained an independent predictor for hepatic decompensation (sHR 1·90 [95% CI 1·21–2·96]; p=0·005; figure 2A; appendix p 9) and hepatocellular carcinoma (5·50 [1·63–15·67]; p=0·005; figure 2B; appendix p 10) after adjustment for liver stiffness on magnetic resonance elastography. Among participants with liver stiffness on magnetic resonance elastography of less than 5 kPa at baseline (n=1312), the 1-year, 3-year, and 5-year risk of incident hepatic decompensation was 1·21% (95% CI 0·41–2·90), 2·70% (1·26–5·06), and 4·48% (2·29–7·77) in participants with type 2 diabetes, and 0·23% (0·05–0·80), 0·94% (0·42–1·88), and 1·39% (0·67–2·58) in participants without type 2 diabetes (p=0·0038 for 5-year risk). Among participants with liver stiffness on magnetic resonance elastography of 5 kPa or higher at baseline (n=425), the 1-year, 3-year, and 5-year risk of incident hepatic decompensation was 6·45% (95% CI 3·77–10·12), 14·45% (9·90–19·81), and 27·96% (20·42–35·97) in participants with type 2 diabetes versus 5·62% (2·75–9·96), 13·22% (8·05–19·70), and 16·99% (10·67–24·56) in participants without type 2 diabetes (p=0·13 for 5-year risk). Type 2 diabetes was an independent predictor of hepatic decompensation in participants without cirrhosis (sHR 2·48 [95% CI 1·10–5·61]; p=0·029; appendix p 11), but not those with cirrhosis (appendix p 12). Type 2 diabetes was an independent predictor of hepatocellular carcinoma among participants with cirrhosis (5·25 [95% CI 1·12–24·67]; p=0·036; appendix p 13), but not those without cirrhosis (appendix p 14); however, these analyses were limited by the small number of events in participants without cirrhosis.

We assessed the impact of HbA_1c_ as a predictor for hepatic decompensation, stratified by type 2 diabetes status, and a similar trend was observed in participants with type 2 diabetes (1·21 [95% CI 0·99–1·49]; p=0·066; appendix p 15), but not in those without type 2 diabetes (1·40 [0·85–2·30]; p=0·19; appendix p 16). We included sex in the multivariable models and determined consistent results with the main analyses (appendix pp 17-19).

## Discussion

In this meta-analysis of individual participant-level data from six international centres, we determined that type 2 diabetes was strongly associated with hepatic decompensation in NAFLD after accounting for appropriate competing risks. The risk of hepatic decompensation was significantly higher in participants with type 2 diabetes than participants without type 2 diabetes. Type 2 diabetes remained an independent predictor of hepatic decompensation after adjustment for multiple confounders and baseline liver stiffness. A higher HbA_1c_ was independently associated with hepatic decompensation after adjustment for confounders. Additionally, type 2 diabetes was associated with a significantly higher risk of hepatocellular carcinoma development and was an independent predictor of hepatocellular carcinoma development after adjustment for confounding factors and baseline liver stiffness.

A prospectively recruited cohort of patients aged 50 years and older with type 2 diabetes showed that the prevalence of advanced liver fibrosis was 14%, although the study was cross-sectional and did not evaluate long-term outcomes.^17^ A study of 447 patients with NAFLD and paired biopsies determined a faster fibrosis progression rate in patients with type 2 diabetes than those without type 2 diabetes, which is likely to contribute to the higher risk of decompensation and hepatocellular carcinoma in type 2 diabetes.^46^ The findings of a previous study of 132 patients with NAFLD showed that type 2 diabetes was independently associated with mortality related to liver disease.^47^ The current study builds on these data by providing high-level evidence that patients with type 2 diabetes are at higher risk for liver-related events. An individual participant data meta-analysis on the risk of liver-related outcomes in participants with NAFLD with and without type 2 diabetes has not been reported, and the current study fills this knowledge gap.

To our knowledge, this is the first pooled analysis of individual participant-level data to assess the risk of hepatic decompensation in patients with type 2 diabetes versus patients without type 2 diabetes. Strengths of our study include the use of individual participant-level data, a large sample size, and ethnically diverse participants from six international centres. However, the study had limitations. The data were retrospectively collected and hence might be subject to bias associated with retrospective studies. There could be unmeasured confounders, such as a family history of cirrhosis, which were not accounted for in the multivariable analyses. Moreover, the data were collected at tertiary centres, which might have introduced a degree of selection bias for participants who were at higher risk of decompensation. We were unable to provide longitudinal data for HbA_1c_ to determine if a reduction in HbA_1c_ was associated with a reduced risk of decompensation. We did not have data on the effect of type 2 diabetes treatment on hepatic decompensation. The follow-up time was modest, and future studies with longer follow-ups might be helpful. The search was also restricted to studies in English, which could have introduced selection bias.

These data have important implications for clinical practice. Type 2 diabetes, and inadequate glycaemic control, were associated with a higher risk of hepatic decompensation and hepatocellular carcinoma in participants with NAFLD. These data highlight the importance of ensuring comparable proportions of participants with type 2 diabetes in the treatment and control groups of clinical trials in NAFLD. These findings indicate the need for a concerted global effort to reduce the morbidity of NAFLD associated with type 2 diabetes.

People with type 2 diabetes have a significantly higher risk of hepatic decompensation and hepatocellular carcinoma than people without type 2 diabetes. Suboptimal glycaemic control is associated with a higher risk of hepatic decompensation and hepatocellular carcinoma. The higher risk of hepatic decompensation and hepatocellular carcinoma in people with type 2 diabetes should be considered when designing clinical trials in NAFLD. These data serve as a call to action to prevent type 2 diabetes and reduce the growing burden of NAFLD and NAFLD-related hepatocellular carcinoma.

## Data sharing

Individual participant data will not be made available to maintain participant confidentiality.

## Supplementary Material

supp

## Figures and Tables

**Table 1.** Baseline characteristics of participants

Empty Cell	Empty Cell	Overall (n=2016)	Participants without type 2 diabetes (n=1280)	Participants with type 2 diabetes (n=736)	p value
**Demographic characteristics**					
Age, years		57·8 (14·2)	55·0 (14·9)	62·8 (11·4)	<0·0001
Sex					
	Female	1074 (53·3%)	673 (52·6%)	401 (54·5%)	0·41
	Male	942 (46·7%)	607 (47·4%)	335 (45·5%)	..
BMI, kg/m^2^		31·3 (7·4)	31·0 (7·4)	31·9 (7·5)	0·011
Race					
	White	1184 (58·7%)	786 (61·4%)	398 (54·1%)	<0·0001
	Hispanic	161 (8·0%)	89 (6·9%)	72 (9·8%)	..
	Asian	605 (30·0%)	353 (27·6%)	252 (34·2%)	..
	Other	66 (3·3%)	52 (4·1%)	14 (1·9%)	..
**Biochemical profile**					
HbA_1c_, %		6·2% (5·6 to 7·2)	5·8% (5·4 to 6·2)	6·8% (6·1 to 7·8)	<0·0001
HbA_1c_, mmol/mol		44 (38 to 55)	40 (36 to 44)	51 (43 to 62)	<0·0001
Aspartate aminotransferase, U/L		40·0 (28 to 59)	38·0 (27 to 57)	45·0 (30 to 65)	<0·0001
Alanine aminotransferase, U/L		45·0 (28 to 75)	46·0 (28 to 76)	44·0 (30 to 70)	0·64
Alkaline phosphatase, U/L		109·0 (76 to 228)	103·0 (73 to 206)	124·0 (84 to 267)	<0·0001
Total bilirubin, mg/dL		10·3 (6·8 to 15·4)	10·3 (6·8 to 15·4)	10·3 (6·8 to 16·9)	0·87
Albumin, g/L		43 (39 to 45)	43 (40 to 46)	42 (38 to 44)	<0·0001
Triglycerides, mg/dL		1·6 (1·2 to 2·3)	1·6 (1·1 to 2·2)	1·8 (1·3 to 2·4)	<0·0001
HDL, mg/dL		1·2 (0·9–1·5)	1·2 (1·0 to 1·5)	1·2 (1·0 to 1·4)	<0·0001
LDL, mg/dL		2·7 (2·1 to 3·4)	2·8 (2·2 to 3·5)	2·6 (2·0–3·3)	0·0004
Platelet count, 10^9^/L		197 (142 to 253)	210 (153 to 266)	172 (123·5 to 227)	<0·0001
International normalised ratio		1·02 (0·99 to 1·10)	1·00 (0·99 to 1·1)	1·05 (1 to 1·11)	0·0056
**Clinical scores**					
Fibrosis-4 index		1·83 (1·11 to 3·23)	1·54 (0·96 to 2·54)	2·44 (1·56 to 4·07)	<0·0001
NAFLD fibrosis score		−0·59 (−1·97 to 0·90)	−1·34 (−2·52 to −0·20])	0·78 (−0·35 to 1·98)	<0·0001
MELD score		7·0 (6 to 9)	7·0 (6 to 9)	7·0 (6 to 9)	0·19
**Imaging**					
Magnetic resonance elastography, kPa		4·14 (2·18)	3·59 (1·82)	5·10 (2·42)	<0·0001
Liver stiffness, kPa					
	<5	1485 (73·7%)	1070 (83·6%)	415 (56·4%)	<0·0001
	≥5	531 (26·3%)	210 (16·4%)	321 (43·6%)	..

Data are mean (SD), n (%), or median (IQR). HbA_1c_=glycated haemoglobin. NAFLD=non-alcoholic fatty liver disease.

**Table 2.** Predictors of incident hepatic decompensation, accounting for death without hepatic decompensation as a competing risk

Empty Cell	Univariable sHR (95% CI)	p value	Multivariable sHR (95% CI)	p value
Age (years)	1·05 (1·03–1·06)	<0·0001	1·05 (1·03–1·07)	<0·0001
BMI (kg/m^2^)	1·03 (1·00–1·05)	0·027	1·03 (1·00–1·06)	0·058
Race (White *vs* non-White)	1·64 (1·05–2·56)	0·029	1·82 (1·09–3·04)	0·022
Presence of type 2 diabetes (*vs* no type 2 diabetes)	3·29 (2·21–4·90)	<0·0001	2·15 (1·39–3·34)	0·0006

Incident hepatic decompensation was defined as ascites, hepatic encephalopathy, or variceal bleeding. sHR=subdistribution hazard ratio.

**Table 3.** Predictors of incident hepatocellular carcinoma, accounting for death without hepatocellular carcinoma as a competing risk

Empty Cell	Univariable sHR (95% CI)	p value	Multivariable sHR (95% CI)	p value
Age (years)	1·07 (1·03–1·11)	0·0006	1·05 (1·01–1·09)	0·029
BMI (kg/m^2^)	0·96 (0·90–1·03)	0·27	0·99 (0·91–1·08)	0·77
Race (White vs non-White)	0·44 (0·19–1·05)	0·064	0·65 (0·23–1·84)	0·42
Presence of type 2 diabetes (*vs* no type 2 diabetes)	7·72 (2·61–22·87)	0·0002	5·34 (1·67–17·09)	0·0048

sHR=subdistribution hazard ratio.

## Research in context

### Evidence before this study

Data regarding the long-term risk of hepatic decompensation in people with non-alcoholic fatty liver disease (NAFLD) and type 2 diabetes are scarce. We searched PubMed for systematic reviews published from database inception until April 24, 2023, using the search terms “nonalcoholic fatty liver disease” and “diabetes mellitus”, but did not identify any individual participant-level data meta-analysis assessing the risk of hepatic decompensation and hepatocellular carcinoma in people with and without type 2 diabetes.

### Added value of this study

This meta-analysis of individual participant-level data from participants with NAFLD from six international sites provides high-level evidence that people with type 2 diabetes are at substantially higher risk of hepatic decompensation and hepatocellular carcinoma, compared to people without type 2 diabetes. The association between type 2 diabetes, hepatic decompensation, and hepatocellular carcinoma remained consistent even after adjusting for baseline liver stiffness. This study determined that suboptimal glycaemic control is associated with a higher risk of hepatic decompensation and hepatocellular carcinoma.

### Implications of all the available evidence

These data highlight the importance of ensuring comparable proportions of participants with type 2 diabetes in the treatment and control arms of clinical trials in NAFLD. Urgent measures are required to prevent type 2 diabetes to slow the growing burden of NAFLD and NAFLD-related hepatocellular carcinoma.
