# Supplementary material for: Type 2 diabetes, hepatic decompensation, and hepatocellular carcinoma in patients with non-alcoholic fatty liver disease: an individual participant-level data meta-analysis
Source: Lancet Gastroenterol Hepatol. Author manuscript; Available in PMC 2024 Jan 26. (PMC10812844; doi:10.1016/S2468-1253(23)00157-7)

THE LANCET
Gastroenterology & Hepatology

Supplementary appendix

This online publication has been corrected. The corrected version first appeared at thelancet.com/gastrohep on July 17, 2023

This appendix formed part of the original submission and has been peer reviewed. We post it as supplied by the authors.

Supplement to: Huang DQ, Noureddin N, Ajmera V, et al. Type 2 diabetes, hepatic decompensation, and hepatocellular carcinoma in patients with non-alcoholic fatty liver disease: an individual participant-level data meta-analysis. *Lancet Gastroenterol Hepatol* 2023; published online July 4. https://doi.org/10.1016/S2468-1253(23)00157-7.

Supplementary Appendix

Search strategy details

Ovid MEDLINE(R) 1946 to April 24, 2023 and Epub Ahead of Print, In-Process & Other Non-Indexed Citations and Ovid MEDLINE(R) Daily, EBM Reviews - Cochrane Central Register of Controlled Trials <March 2023>, EBM Reviews - Cochrane Database of Systematic Reviews <2005 to April 24, 2023>, Embase <1974 to 2023 April 24>

1	magnetic resonance elastography/ or elasticity imaging techniques/ or magnetic resonance elastograpy.mp. or MRE.mp.		33790
2	nonalcoholic fatty liver/ or Non-alcoholic Fatty Liver Disease/ or nonalcoholic fatty liver.mp. or non-alcoholic fatty liver.mp. or NAFLD.mp.		111633
3	(stiff or stiffness).mp.		257576
4	1 and 2 and 3		1575
5	limit 4 to english language [Limit not valid in CDSR; records were retained]		1553
6	limit 5 to "all adult (19 plus years)" [Limit not valid in CCTR,CDSR,Embase; records were retained]		1335
7	limit 6 to (adult <18 to 64 years> or aged <65+ years>) [Limit not valid in Ovid MEDLINE(R),Ovid MEDLINE(R) Daily Update,Ovid MEDLINE(R) PubMed not MEDLINE,Ovid MEDLINE(R) In-Process,Ovid MEDLINE(R) Publisher,CCTR,CDSR; records were retained]		913
8	limit 7 to embase status [Limit not valid in Ovid MEDLINE(R),Ovid MEDLINE(R) Daily Update,Ovid MEDLINE(R) PubMed not MEDLINE,Ovid MEDLINE(R) In-Process,Ovid MEDLINE(R) Publisher,CCTR,CDSR; records were retained]		687
9	remove duplicates from 8		566
10	limit 9 to human [Limit not valid in CCTR,CDSR; records were retained]		566

SCOPUS

1	TITLE-ABS-KEY ((“magnetic AND resonance AND elastography” OR mre) AND (“nonalcoholic AND fatty AND liver” OR “non-alcoholic AND fatty AND liver” OR nafld) AND (stiff OR stiffness))	73
2	(LIMIT-TO (LANGUAGE , "English"))	73
3	(LIMIT-TO (DOCTYPE , "ar"))	60
4	AND (adult)	55

PubMed, 272 results (limits: English language, adult, human)

(("Elasticity Imaging Techniques"[Mesh] OR Magnetic Resonance Elastography [tiab] OR MRE [tiab]) AND ("Non-alcoholic Fatty Liver Disease"[Mesh] OR Non-alcoholic Fatty Liver [tiab] nonalcoholic fatty liver [tiab] OR NAFLD [tiab]) AND (stiff [tiab] OR stiffness [tiab])) NOT letter [pt] NOT editorial [pt] NOT review [pt]

Web of Science from 1975 to 2023 April 24 : 287 references

1	ALL=(magnetic resonance elastography or elasticity imaging techniques or magnetic resonance elastograpy or MRE)	10,822
2	ALL=(nonalcoholic fatty liver or Non-alcoholic Fatty Liver Disease or nonalcoholic fatty liver or non-alcoholic fatty liver or NAFLD)	44,123
3	ALL=(stiff or stiffness)	256,336
4	#1 AND #2 AND #3	290
5	LA = (English)	55,512,684
6	#4 AND #5	287

1180 total references, 418 duplicates found in EndNote

762 total references in EndNote

Supplementary Table 1: Risk of bias assessment among published studies

Study	Risk of bias				Applicability concerns		
	Patient Selection	Index test	Reference standard	Flow and timing	Patient selection	Index test	Reference standard
Matsui	Low	High (MRE threshold was not prespecified)	Unclear	Low	Low	Low	Low
Han	High (Patients with weight gain more that 5% within 6 months of MRE were excluded)	High (MRE threshold was not prespecified)	Low	Low	High (We do not have this exclusion criteria in our systematic review question)	Low	Low
Gidener	Low	Low	Unclear (it was not reported whether the outcomes assessment was blinded to the MRE results)	High (patients with less than 30 days of follow up were excluded from analysis)	Low	Low	Low
Tamaki	High (patients with follow up less than six months were excluded)	Unclear (it was unclear if the index test results were interpreted without knowledge of the results of the standard test)	Unclear	Unclear	Low	Low	Unclear
Ajmera	Low	Low	Unclear (it was not reported whether the outcomes assessment was blinded to the MRE results)	Low	Low	Low	Low

Abbreviations: MRE, magnetic resonance elastography

Supplemental Table 2. Factors associated with hepatic decompensation (ascites, hepatic encephalopathy, or variceal bleeding) at baseline

	**Univariable OR**	**P-value**	**Multivariable OR**	**P-value**
Age (years)	1.02 (1.01-1.04)	0.0035	1.01 (0.99-1.03)	0.1136
BMI (kg/m^2)	1.03 (1.00-1.05)	0.0279	1.00 (0.97-1.03)	0.9865
Race/ethnicity (White versus non-White)	3.73 (2.62-6.16)	<.0001	4.65 (2.65-8.15)	<.0001
Presence of T2DM (versus no T2DM)	2.95 (2.00-4.36)	<.0001	3.08 (1.98-4.78)	<.0001

Abbreviations: OR, odds ratio; BMI, body mass index; T2DM, type 2 diabetes mellitus

Supplemental Table 3. HbA1c as a predictor of incident hepatic decompensation (ascites, hepatic encephalopathy, or variceal bleeding), accounting for death without hepatic decompensation as a competing risk

	**Univariable sHR**	**P-value**	**Multivariable sHR**	**P-value**
Age (years)	1.05 (1.03-1.06)	<·0001	1·06 (1·03-1·08)	<·0001
BMI (kg/m^2)	1·03 (1·00-1·05)	0·027	1·02 (0·98-1·06)	0·42
Race/ethnicity (White versus non-White)	1·64 (1·05-2·56)	0·029	1·50 (0·79-2·85)	0·22
HbA1c (per 1-unit increase)	1·30 (1·13-1·49)	0·0002	1·31 (1·10-1·55)	0·0019

Abbreviations: sHR, subdistribution hazard ratio; BMI, body mass index; HbA1c, hemoglobin A1c

Supplemental Table 4. Factors associated with incident development of HCC, with death without HCC as a competing risk

	**Univariable sHR**	**P-value**	**Multivariable sHR**	**P-value**
Age (years)	1.07 (1.03-1.11)	0.0006	1.07 (1.002-1.12)	0.0052
BMI (kg/m^2)	0.96 (0.90-1.03)	0.27	0.95 (0.87-1.04)	0.30
Race/ethnicity (White versus non-White)	0.44 (0.19-1.05)	0.064	0.36 (0.12-1.06)	0.065
HbA1c (per 1-unit increase)	1.21 (1.01-1.44)	0.039	1.32 (1.02-1.71)	0.034

Abbreviations: HCC, hepatocellular carcinoma; sHR, subdistribution hazard ratio; BMI, body mass index; T2DM, type 2 diabetes mellitus

Supplemental Table 5. Predictors of incident hepatic decompensation (ascites, hepatic encephalopathy, or variceal bleeding), accounting for death without hepatic decompensation as a competing risk, adjusted for liver stiffness by MRE

	**Univariable sHR**	**P-value**	**Multivariable sHR**	**P-value**
Liver stiffness by MRE (kPA)	1.39 (1.29-1.49)	<.0001	1.35 (1.25-1.45)	<.0001
Presence of T2DM (versus no T2DM)	3.29 (2.21-4.90)	<.0001	1.90 (1.21-2.96)	0.0050

Abbreviations: MRE, magnetic resonance elastography; sHR, subdistribution hazard ratio; T2DM, type 2 diabetes mellitus

Supplemental Table 6. Predictors of incident HCC, accounting for death without HCC as a competing risk, adjusted for liver stiffness by MRE

	**Univariable sHR**	**P-value**	**Multivariable sHR**	**P-value**
Liver stiffness by MRE (kPA)	1.36 (1.26-1.47)	<.0001	1.28 (1.17-1.39)	<.0001
Presence of T2DM (versus no T2DM)	7·72 (2·61-22·87)	0·0002	5.50 (1.63-15.67)	0.005

Abbreviations: HCC, hepatocellular carcinoma; MRE, magnetic resonance elastography; sHR, subdistribution hazard ratio; T2DM, type 2 diabetes mellitus

Supplemental Table 7. Predictors of incident hepatic decompensation (ascites, hepatic encephalopathy, or variceal bleeding), accounting for death without hepatic decompensation as a competing risk, among participants without cirrhosis (MRE < 5.0 kPa)

	**Univariable SHR**	**P-value**	**Multivariable sHR**	**P-value**
Age (years)	1.07 (1.04-1.10)	<.0001	1.06 (1.03-1.09)	0.0002
BMI (kg/m^2)	0.99 (0.94-1.04)	0.65	0.98 (0.92-1.05)	0.58
Race/ethnicity (White versus non-White)	1.54 (0.60-3.97)	0.37	2.75 (0.99-7.60)	0.052
Presence of T2DM (versus no T2DM)	3.56 (1.61-7.88)	0.0017	2.48 (1.10-5.61)	0.029

Abbreviations: MRE, magnetic resonance elastography; sHR, subdistribution hazard ratio; BMI, body mass index; T2DM, type 2 diabetes mellitus

Supplemental Table 8. Predictors of incident hepatic decompensation (ascites, hepatic encephalopathy, or variceal bleeding), accounting for death without hepatic decompensation as a competing risk, among participants with cirrhosis (MRE ≥ 5.0 kPa)

	**Univariable sHR**	**P-value**	**Multivariable sHR**	**P-value**
Age (years)	1.02 (1.00-1.03)	0.0312	1.03 (1.01-1.05)	0.0038
BMI (kg/m^2)	1.05 (1.03-1.08)	<.0001	1.04 (1.00-1.07)	0.033
Race/ethnicity (White versus non-White)	2.57 (1.56-4.26)	0.0002	2.30 (1.26-4.22)	0.0070
Presence of T2DM (versus no T2DM)	1.43 (0.90-2.27)	0.13	1.00 (0.62-1.62)	0.99

Abbreviations: MRE, magnetic resonance elastography; sHR, subdistribution hazard ratio; BMI, body mass index; T2DM, type 2 diabetes mellitus

Supplemental Table 9. Predictors of incident hepatocellular carcinoma, accounting for death without hepatic decompensation as a competing risk, among participants with cirrhosis (MRE ≥ 5.0 kPa)

	**Univariable SHR**	**P-value**	**Multivariable sHR**	**P-value**
Age (years)	1.05 (1.01-1.10)	0.015	1.04 (0.99-1.09)	0.10
BMI (kg/m^2)	0.96 (0.90-1.04)	0.33	0.97 (0.88-1.07)	0.56
Race/ethnicity (White versus non-White)	0.47 (0.18-1.21)	0.12	0.71 (0.24-2.11)	0.53
Presence of T2DM (versus no T2DM)	6.12 (1.43-26.23)	0.015	5.25 (1.12-24.67)	0.036

Abbreviations: MRE, magnetic resonance elastography; sHR, subdistribution hazard ratio; BMI, body mass index; T2DM, type 2 diabetes mellitus

Supplemental Table 10. Predictors of incident HCC, accounting for death without hepatic decompensation as a competing risk and adjusted for a minimally sufficient set of confounders, among participants without cirrhosis (MRE < 5.0 kPa)

	**Univariable SHR**	**P-value**	**Multivariable sHR**	**P-value**
Age (years)	1.03 (0.94-1.14)	0.41	1.03 (0.93-1.14)	0.59
BMI (kg/m^2)	0.97 (0.90-1.05)	0.46	0.97 (0.83-1.13)	0.71
Race/ethnicity (White versus non-White)	1.06 (0.09-12.48)	0.96	1.46 (0.05-40.49)	0.82
Presence of T2DM (versus no T2DM)	1.24 (0.12-13.00)	0.86	1.12 (0.07-18.89)	0.94

Abbreviations: HCC, hepatocellular carcinoma; MRE, magnetic resonance elastography; sHR, subdistribution hazard ratio; BMI, body mass index; T2DM, type 2 diabetes mellitus

Supplemental Table 11. HbA1c as a predictor of incident hepatic decompensation (ascites, hepatic encephalopathy, or variceal bleeding), accounting for death without hepatic decompensation as a competing risk, among participants with T2DM

	**Univariable sHR**	**P-value**	**Multivariable sHR**	**P-value**
Age (years)	1.02 (1.00-1.04)	0.030	1.04 (1.01-1.07)	0.0084
BMI (kg/m^2)	1.04 (1.00-1.07)	0.029	1.03 (0.99-1.08)	0.19
Race/ethnicity (White versus non-White)	1.90 (1.12-3.23)	0.017	1.21 (0.59-2.52)	0.60
HbA1c (per 1-unit increase)	1.18 (0.98-1.42)	0.080	1.21 (0.99-1.49)	0.066

Abbreviations: T2DM, type 2 diabetes mellitus; sHR, subdistribution hazard ratio; BMI, body mass index; HbA1c, hemoglobin A1c

Supplemental Table 12. HbA1c as a predictor of incident hepatic decompensation (ascites, hepatic encephalopathy, or variceal bleeding), accounting for death without hepatic decompensation as a competing risk, among participants without T2DM

	**Univariable sHR**	**P-value**	**Multivariable sHR**	**P-value**
Age (years)	1.06 (1.04-1.09)	<0.0001	1.08 (1.03-1.13)	0.0009
BMI (kg/m^2)	1.00 (0.97-1.04)	0.84	0.91 (0.81-1.02)	0.092
Race/ethnicity (White versus non-White)	2.07 (0.90-4.76)	0.088	4.22 (0.97-18.36)	0.055
HbA1c (per 1-unit increase)	1.16 (0.69-1.93)	0.58	1.40 (0.85-2.30)	0.19

Abbreviations: HbA1c, hemoglobin A1c; T2DM, type 2 diabetes mellitus; sHR, subdistribution hazard ratio; BMI, body mass index

Supplemental Table 13. Predictors of incident hepatic decompensation (ascites, hepatic encephalopathy, or variceal bleeding), accounting for death without hepatic decompensation as a competing risk, with sex in the model

	**Univariable sHR**	**P-value**	**Multivariable sHR**	**P-value**
Age (years)	1.05 (1.03-1.06)	<0.0001	1.05 (1.03-1.07)	<0.0001
Female sex	1.72 (1.16-2.57)	0.0075	1.36 (0.89-2.07)	0.16
BMI (kg/m^2)	1.03 (1.00-1.05)	0.027	1.02 (0.99-1.05)	0.10
Race/ethnicity (White versus non-White)	1.64 (1.05-2.56)	0.029	1.84 (1.10-3.07)	0.020
Presence of T2DM (versus no T2DM)	3.29 (2.21-4.90)	<0.0001	2.16 (1.39-3.34)	0.0006

Abbreviations: sHR, subdistribution hazard ratio; BMI, body mass index; T2DM, type 2 diabetes mellitus

Supplemental Table 14. HbA1c as a predictor of incident hepatic decompensation (ascites, hepatic encephalopathy, or variceal bleeding), accounting for death without hepatic decompensation as a competing risk, with sex in the model

	**Univariable sHR**	**P-value**	**Multivariable sHR**	**P-value**
Age (years)	1.05 (1.03-1.06)	<0.0001	1.06 (1.03-1.08)	<0.0001
Female sex	1.72 (1.16-2.57)	0.0075	1.28 (0.74-2.21)	0.38
BMI (kg/m^2)	1.03 (1.00-1.05)	0.027	1.02 (0.97-1.06)	0.50
Race/ethnicity (White versus non-White)	1.64 (1.05-2.56)	0.029	1.50 (0.79-2.85)	0.22
HbA1c (per 1-unit increase)	1.30 (1.13-1.49)	0.0002	1.31 (1.10-1.55)	0.0019

Abbreviations: HbA1c, hemoglobin A1c; sHR, subdistribution hazard ratio; BMI, body mass index; T2DM, type 2 diabetes mellitus

Supplemental Table 15. Predictors of incident HCC, accounting for death without HCC as a competing risk, with sex in the model

	**Univariable sHR**	**P-value**	**Multivariable sHR**	**P-value**
Age (years)	1.07 (1.03-1.11)	0.0006	1.05 (1.01-1.10)	0.018
Female sex	0.50 (0.21-1.20)	0.12	0.41 (0.16-1.02)	0.055
BMI (kg/m^2)	0.96 (0.90-1.03)	0.27	1.00 (0.91-1.09)	0.94
Race/ethnicity (White versus non-White)	0.44 (0.19-1.05)	0.06	0.65 (0.22-1.91)	0.44
Presence of T2DM (versus no T2DM)	7.72 (2.61-22.87)	0.0002	5.27 (1.65-16.82)	0.0049

Abbreviations: HCC, hepatocellular carcinoma; sHR, subdistribution hazard ratio; BMI, body mass index; T2DM, type 2 diabetes mellitus

Supplemental Figure 1. Directed acyclic graph summarizing the associations between T2DM, covariates, and the primary outcome of hepatic decompensation. Beige circles represent ancestors of the exposure and outcome, the green circle represents the causal determinant of interest. Dark blue represents the causal path, while black represents potential biasing paths. The final minimally sufficient set of confounders included age, race/ethnicity, and body mass index.

Abbreviations: T2DM, type 2 diabetes mellitus; BMI, body mass index

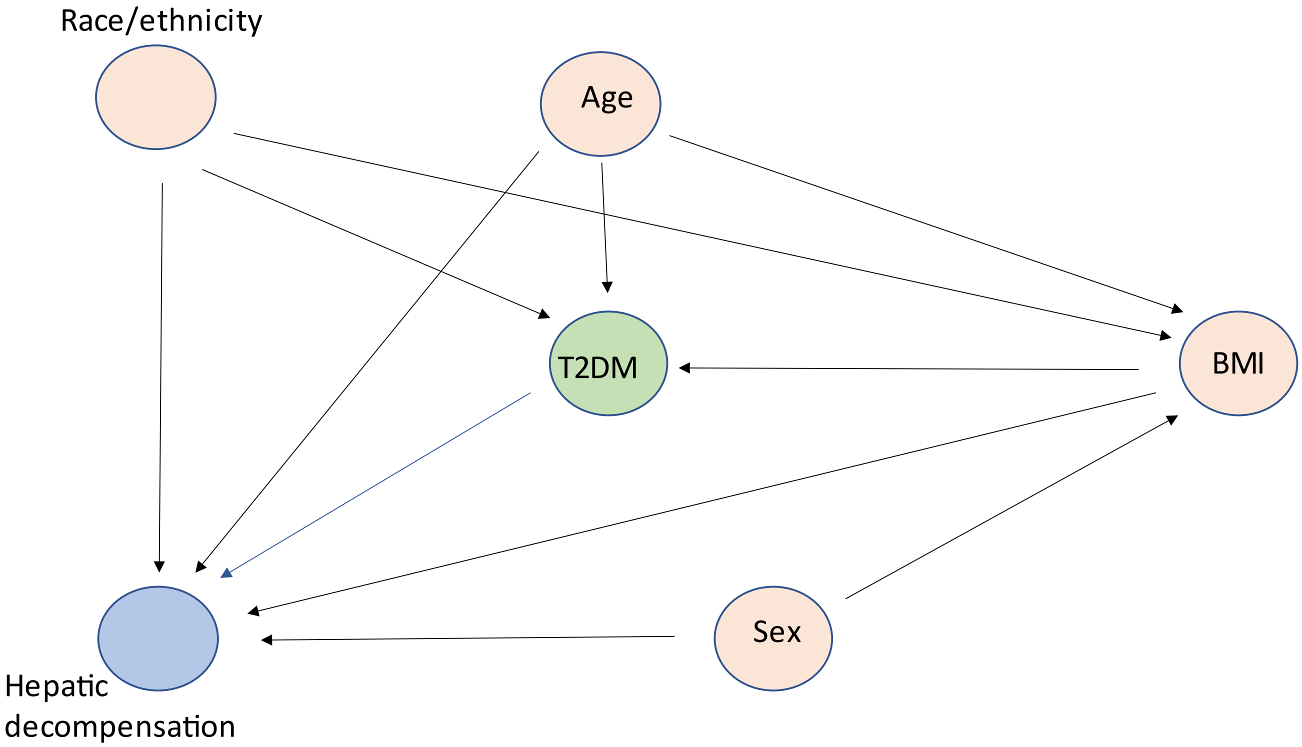

Supplemental Figure 2. Study selection

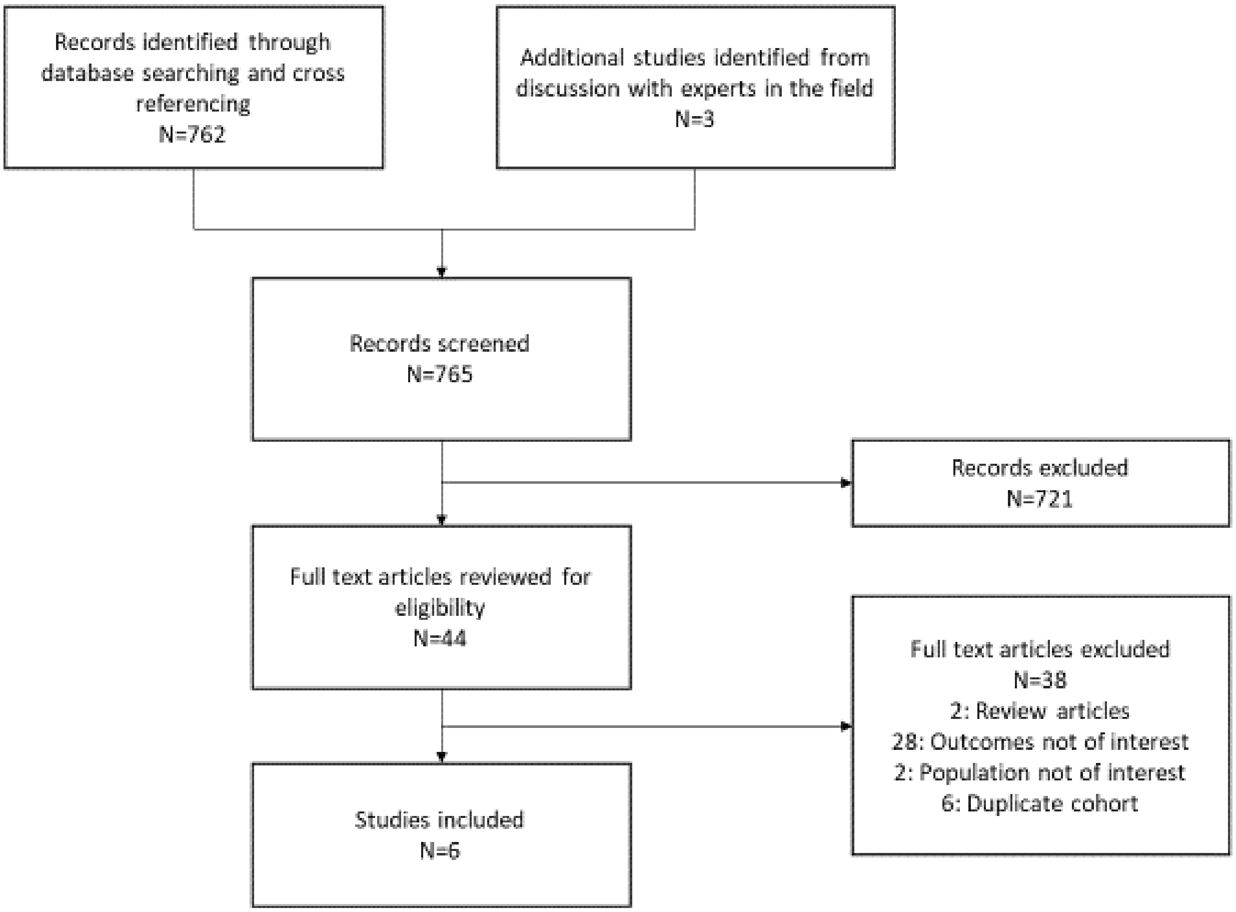

Supplement: supp [file NIHMS1959537-supplement-supp.pdf]
